# Molecular Characterization of Near Full-Length Genomes of Hepatitis B Virus Isolated from Predominantly HIV Infected Individuals in Botswana

**DOI:** 10.3390/genes9090453

**Published:** 2018-09-07

**Authors:** Motswedi Anderson, Wonderful Tatenda Choga, Sikhulile Moyo, Trevor Graham Bell, Tshepiso Mbangiwa, Bonolo Bonita Phinius, Lynnette Bhebhe, Theresa Kibirige Sebunya, Shahin Lockman, Richard Marlink, Anna Kramvis, Max Essex, Rosemary Mubanga Musonda, Jason Tory Blackard, Simani Gaseitsiwe

**Affiliations:** 1 Botswana Harvard AIDS Institute Partnership, Gaborone, Botswana; motswedi.anderson@gmail.com (M.A.); wtchoga@gmail.com (W.T.C.); smoyo@bhp.org.bw (S.M.); mbangiwat@gmail.com (T.M.); bphinius@gmail.com (B.B.P.); lynnettebhebhe@gmail.com (L.B.); shahin.lockman@gmail.com (S.L.); rmarlink@globalhealth.rutgers.edu (R.M.); messex@hsph.harvard.edu (M.E.); rmusonda@bhp.org.bw (R.M.M.); 2 Department of Biological Sciences, Faculty of Science, University of Botswana, Gaborone, Botswana; sebunyat@gmail.com; 3 Department of Immunology and Infectious Diseases, Harvard T.H. Chan School of Public Health, Boston, MA 02138, USA; 4 Hepatitis Virus Diversity Research Unit (HVDRU), Department of Internal Medicine, School of Clinical Medicine, Faculty of Health Sciences, University of the Witwatersrand, Johannesburg 2050, South Africa; TrevorGrahamBell@gmail.com (T.G.B.); Anna.Kramvis@wits.ac.za (A.K.); 5 Faculty of Allied Health Sciences, University of Botswana, Gaborone, Botswana; 6 Rutgers Global Health Institute, Robert Wood Johnson Medical School, Rutgers University, New Brunswick, NJ 08901, USA; 7 College of Medicine, University of Cincinnati, Cincinnati, OH 45627, USA; jason.blackard@uc.edu

**Keywords:** HBV, mutations, occult hepatitis B, chronic hepatitis B, Botswana, Africa

## Abstract

The World Health Organization plans to eliminate hepatitis B and C Infections by 2030. Therefore, there is a need to study and understand hepatitis B virus (HBV) epidemiology and viral evolution further, including evaluating occult (HBsAg-negative) HBV infection (OBI), given that such infections are frequently undiagnosed and rarely treated. We aimed to molecularly characterize HBV genomes from 108 individuals co-infected with human immunodeficiency virus (HIV) and chronic hepatitis B (CHB) or OBI identified from previous HIV studies conducted in Botswana from 2009 to 2012. Full-length (3.2 kb) and nearly full-length (~3 kb) genomes were amplified by nested polymerase chain reaction (PCR). Sequences from OBI participants were compared to sequences from CHB participants and GenBank references to identify OBI-unique mutations. HBV genomes from 50 (25 CHB and 25 OBI) individuals were successfully genotyped. Among OBI participants, subgenotype A1 was identified in 12 (48%), D3 in 12 (48%), and E in 1 (4%). A similar genotype distribution was observed in CHB participants. Whole HBV genome sequences from Botswana, representing OBI and CHB, were compared for the first time. There were 43 OBI-unique mutations, of which 26 were novel. Future studies using larger sample sizes and functional analysis of OBI-unique mutations are warranted.

## 1. Introduction

Hepatitis B virus (HBV) remains a major global health problem, with approximately 257 million people chronically infected [1]. Viral hepatitis is now the seventh leading cause of death worldwide, with a 63% mortality increase to 1.45 million from 1990 to 2013 [2]. HBV accounts for most viral hepatitis associated deaths [2]. It is endemic in Africa and Western Pacific, with about 6% chronic infections in adults [3]. In Botswana, the prevalence of HBV is between 3.8% and 10.6% in human immunodeficiency virus (HIV)-infected individuals [4,5,6,7].

HBV is a DNA virus belonging to the family *Hepadnaviradae* that consists of a partially double stranded 3.2 kb genome arranged in four partially overlapping open reading frames (ORFs) [8]. The polymerase (Pol) ORF encodes for the polymerase enzyme, which is responsible for DNA priming and reverse transcription during replication [9]. The pre-surface1/pre-surface2/surface (preS1/preS2/S) ORF codes for the large, middle, and small hepatitis B surface antigen (HBsAg) proteins, respectively, which are used to diagnose HBV, contain several B and T cell epitopes, and are utilized for attaching to hepatocytes [10]. The precore/core (preC/C) ORF encodes for hepatitis e antigen (HBeAg)—an indicator for viral replication and an immune regulator [9]—and hepatitis B core antigen (HBcAg)—the capsid protein. The X ORF codes for the X trans-activator protein [11].

Hepadnaviruses are unique in that they replicate via a pregenomic RNA intermediate, which is reverse transcribed by the polymerase, an enzyme with no proof-reading capabilities [12]. This leads to nucleotide misincorporations during replication and accounts for the significant HBV heterogeneity observed within individuals [13,14]. To date, HBV has been classified into at least nine genotypes (A–I) and a putative 10th genotype J according to nucleotide divergence of >7.5% at whole genome level [15,16,17,18]. Several genotypes have been divided further into subgenotypes based on intergroup nucleotide divergence of 4–8% [18].

Routine screening for HBV is performed by detection of HBsAg. However, this approach misses occult hepatitis B infections (OBI), defined as the presence of HBV DNA in HBsAg negative participants [19]. True OBI is characterized by low HBV DNA levels (<200 IU/mL) [19]. OBI is an important clinical entity that poses diagnostic challenges and, transmissible via blood donations, mother to child transmission, close contact with infected individuals, sexual transmission, and organ transplantation [20,21,22,23,24,25,26]. In addition, end stage clinical complications associated with OBI include cirrhosis and hepatocellular carcinoma (HCC) [27,28,29]. The risk of an individual with an HBsAg-negative, HBV DNA-positive individual infection is not negligible. In a recently completed study in South Africa, it was shown that the risk of a HBsAg-negative, HBV DNA-positive individual (with or without anti-HBc) developing HCC was 5.10 (2.06–12.62) compared to a risk of 34.48 (16.26–73.13) in HBsAg-positive individuals (with or without anti-HBc), adjusted for age group, sex, HCV serostatus, country of birth, and HIV status [30].

Several mechanisms may lead to OBI. These include host factors [31,32]; coinfection with other viruses like hepatitis C virus (HCV) and HIV [33,34]; and viral diversity including point mutations, deletions, and insertions [35,36,37]. The majority of studies that identified occult-associated mutations focused on specific genomic regions, such as the preS1/preS2/S ORF. Mutations within this region may affect HBsAg detection and secretion [37,38,39,40,41,42]. However, mutations in the Pol, X, and preC/C ORFs may also play a role in the OBI phenotype [36,43,44].

There is a call by World Health Organization to eliminate HBV and HCV infections as a public health problem by 2030 [45]. To achieve this, robust sequence data of HBV isolated from chronic hepatitis (CHB) and OBI are necessary. However, there are very few studies that evaluated full-length OBI genomes owing to the low viremia that is characteristic of OBI [35]. Furthermore, some studies did not have controls such as CHB participants from the same population to accurately identify occult-associated mutations [46,47]. Thus, the objective of this study was to conduct robust molecular characterization of nearly full-length HBV genomes from individuals with CHB and OBI in Botswana.

## 2. Materials and Methods

### 2.1. Population

This was a cross-sectional study comprising 108 known HBV-infected individuals (both CHB and OBI) from previous studies conducted at the Botswana Harvard AIDS Institute Partnership (BHP) in Gaborone. All available HBV positive samples from various cohorts were utilized to maximize the number of genomes available for analysis. One hundred participants—72 with OBI and 28 with CHB—were included from the Botswana National Evaluation Models of HIV Care (Bomolemo) study, which enrolled HIV positive individuals initiating highly active antiretroviral therapy (HAART) between 2009 and 2012. The Bomolemo study evaluated the efficacy and tolerability of tenofovir and emtricitabine (Truvada™) as the nucleoside reverse transcriptase inhibitor (NRTI) backbone as first-line HAART for adults in Botswana. The HBV screening for the Bomolemo cohort was previously described in detail [6,48]. An additional eight CHB participants—four HIV-positive and four HIV-negative pregnant women—were included from a study comparing the effects of HIV and ARV exposure on child health and neurodevelopment (Tshipidi study) [49]. HBV screening for participants from this cohort was previously described [50].

### 2.2. Ethical Considerations

The study was approved by the University of Botswana Institute Review Board and the Human Research Development Committee at the Botswana Ministry of Health. The Office of Human Research Administration at the Harvard T.H. Chan School of Public Health approved the Tshipidi and Bomolemo studies. Ethics permit number: PPME 13/1811V (318).

### 2.3. Extraction of Plasma DNA

DNA was extracted from 1 mL of plasma using QiAamp Ultrasense virus kit according to the manufacturer’s protocol (Qiagen, Hilden, Germany). An elution volume of 30 μL was used. The extracted DNA was either used immediately for polymerase chain reaction (PCR) or stored at −70 °C until used.

### 2.4. Amplification and Sequencing

Full-length genomes were amplified in two fragments. A 3 kb fragment was amplified by nested PCR using PrimeSTAR^®^ GXL DNA Polymerase kit (Takara Bio Inc., Shiga, Japan) with some modifications. The master mix was composed of 5 µL of 5X PrimeSTAR GXL Buffer containing 5 mM Mg^2+^, 0.5 µL of PrimeSTAR GXL DNA Polymerase (1.25 U/µL), 0.5 µL of 5 µm P1 primer, 0.5 µL of 5 µm P2 primer, 1.0 µL of DNA extract, and 17.5 µL of dH_2_O to make up a 25 µL reaction. The primers used were P1 and P2 for first round, whereas second-round primers were P3WRS and P4WRS [51,52], Table 1. The cycling conditions were 35 cycles of denaturation at 98 °C for 10 s, annealing at 50 °C for 15 s, and extension at 68 °C for 9 min. The second-round cycling conditions were similar to those of first round except for annealing temperature set at 55 °C for 15 s and extension temperature at 68 °C for 4 min. The remaining 276 base-pair (bp) of the preC/C region was amplified using One Step superscript III (Invitrogen, Waltham, MA, USA) protocol with minor modifications. A semi-nested PCR was performed using KU1 and MA3 as first round primers and MA3 and KU2 as second-round primers [53,54], Table 1. The 3 kb and 276 bp amplicons were visualized in 1% and 2% ethidium bromide stained gels, respectively. PCR products were purified using QiAquick PCR Purification (Qiagen, Hilden, Germany), and sequencing clean-up was done using ZR DNA Sequencing Clean up Kit (Zymo, Irvine, CA, USA) according to the manufacture’s protocol. Direct sequencing was then performed on an ABI 3130xl genetic analyzer (Applied Biosystems, Foster City, CA, USA) using Big Dye sequencing chemistry. The primers used for sequencing are shown in Table 1. Sequences were submitted to National Center for Biotechnology Information (NCBI) GenBank under the accession numbers MH464807 to MH464856.

### 2.5. Phylogenetic Analysis

Phylogenetic trees were constructed utilizing a Bayesian Markov chain Monte Carlo (MCMC) in the Bayesian Evolutionary Analysis by Sampling Trees (BEAST) v1.8.2 (BEAST Developers) [57] program with a chain length of 100,000,000 and sampling every 10,000 generations. The analysis utilized an uncorrelated log-normal relaxed molecular clock, the Hasegawa, Kishino, and Yano (HKY) model, and the general time reversible model with gamma distributed rates of variation among sites and a proportion of invariable sites (GTR+G+I). Tracer v1.7 (BEAST Developers) [57] was used to visualize results and confirm chain convergence. Every parameter had an effective sample size (ESS) > 500 implying sufficient sampling. Tree Annotator v1.7.3 (BEAST Developers) [57] was utilized to choose the maximum clade credibility tree after a 10% burn-in. Posterior probabilities > 90% were deemed statistically significant. Trees for subgenotype A1 and D3 sequences from this study and the respective GenBank references for the whole surface region were constructed. The S ORF was used to determine the clustering of Botswana strains relative to other African HBV sequences, because there are very few whole genome HBV sequences from Africa except for South Africa.

### 2.6. HBV Genotype Recombination Analysis

To evaluate potential recombination, study sequences were compared to GenBank references A-H utilizing Simplot software v3.5.1 (Ray, S.C, Baltimore, MD, USA) with a step size of 20 bp and 200 bp window size [58]. The neighbor-joining method (NJ) was used to conduct a bootscan analysis utilizing 1000 bootstrap replicates and an 80% threshold.

### 2.7. Analysis of Immune Selection Pressure, Signature Amino Acids, and Escape Mutations

DataMonkey was used to approximate the rates of nonsynonymous (dN) and synonymous (dS) substitutions using fixed effects likelihood (FEL) [59]. For this analysis, codons for each ORF (S, preS1, preS2, Pol, X, core) were analyzed individually according to HBV status (either CHB or OBI). Codon positions were numbered from the beginning of each open reading frame. The preC region could not be amplified from several OBI participants; hence, it was excluded from this analysis. The viral epidemiology signature pattern analysis (VESPA) was utilized to search for signature amino acids (aa) in HBV sequences isolated from OBI participant’s sequences compared to those isolated from CHB participants for each ORF based on subgenotype [60]. Escape mutations were identified using the online tool Geno2pheno available at https://hbv.geno2pheno.org/. Escape mutations previously reported in the literature were also searched for manually in BioEdit.

### 2.8. Mutational Analysis

Genomes of HBV were aligned with GenBank references using ClustalX v2.1 (Higgins D., Sievers F., Dineen D., Wilm A, Dublin, Ireland). The subgenotype A1 sequences were aligned with 107 full-length subgenotype A1 references, while the subgenotype D3 sequences were aligned with 85 full-length subgenotype D3 references. The references used were HBV sequences isolated from CHB participants, which were extracted from online curated GenBank alignments [61]. All subgenotypes’ A1 and D3 whole genome sequences available at that time (January 2017) were included. The sequences were then trimmed to the same length in BioEdit v7.2.5 (Hall T., Carlsbad, CA, USA). Subsequently, Babylon Translator was utilized to extract each ORF (Pre S1, Pre S2, S, X, PreC, core, Pol) and the sequences translated into aa [62]. To identify potential OBI-unique mutations, sequences from CHB participants were first compared with the OBI sequences for each respective subgenotype to identify potential OBI unique mutations and then compared to the GenBank reference sequences. To avoid polymorphisms that may represent subgenotype differences, sequences were compared at a subgenotype level [63,64]. Mutations that were unique to HBV isolated from OBI participants without appearing in any sequences from CHB patients or references from CHB were classified as occult-unique mutations [41,47,65,66,67,68,69].

## 3. Results

There were no statistically significant differences at baseline in terms of CD4^+^ T cell count, HIV viral load, liver enzymes, Fibrosis 4 (a noninvasive measure of liver scarring), or other clinical parameters between the CHB, OBI, and the HBV negative participants as reported in detail elsewhere [48,50]. The HBV viral loads were low in the OBI group with a median of 57.4 copies per mL versus 31,600 copies per mL in the CHB group as reported elsewhere [48]. HBV antigens results (HBsAg and HBeAg) and HBV antibodies (anti-HBc and anti-HBs) have been reported in detail elsewhere [48,50].

**Figure 1:**
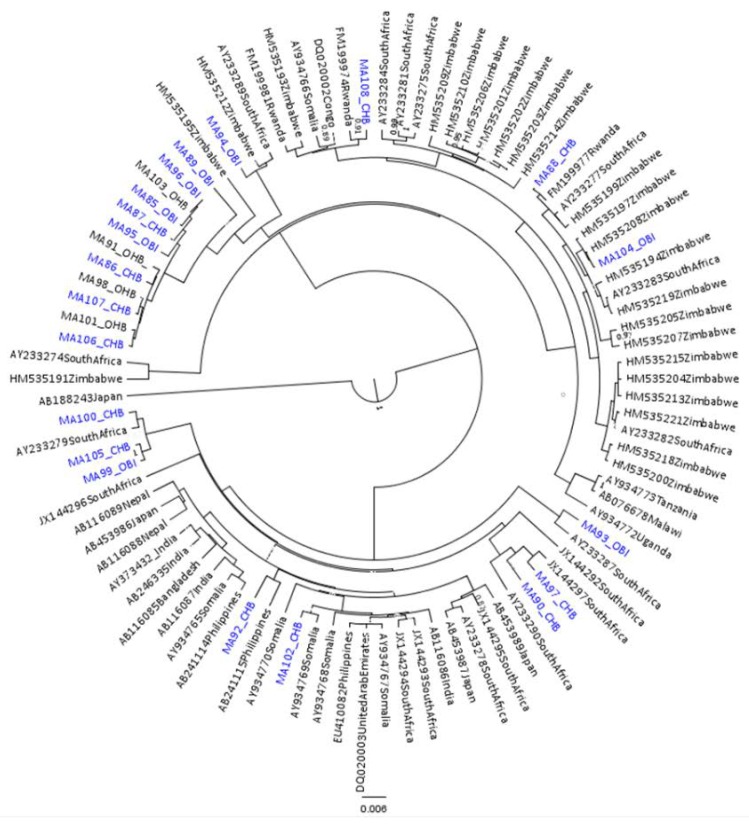
A phylogenetic tree of the whole surface region (nucleotide (nt) 2854–835 from EcoRI site) of subgenotype A1 hepatitis B virus (HBV) sequences generated by Bayesian Evolutionary Analysis by Sampling Trees (BEAST). Strains from Botswana sequenced in the present study are shown in blue, while reference sequences are shown in black. Reference strains are designated by their accession number and country of origin, whereas Botswana sequences are designated by MA followed by a number and either CHB (for chronic HBV strains) or OBI (for occult HBV strains).

**Figure 2:**
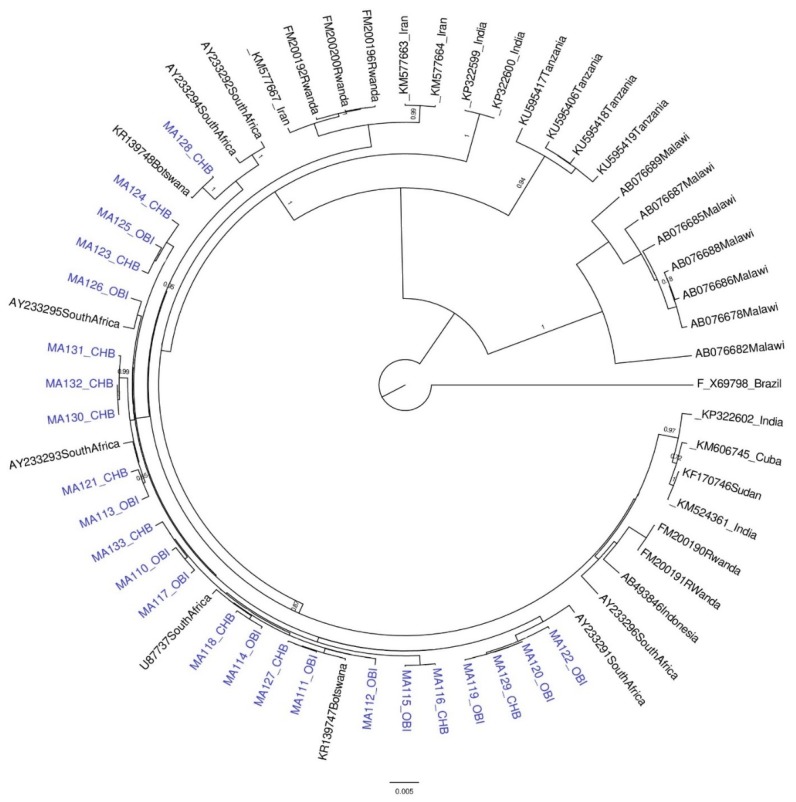
A phylogenetic tree of the whole surface region (nt 2854–835 from *Eco*RI site) of HBV subgenotype D3 generated by BEAST. Botswana sequences are shown in blue, whereas reference sequences are in black. Reference strains are designated by their accession number and country of origin, whereas Botswana sequences are designated by MA followed by a number and either CHB or OBI.

Of the 108 participants, HBV genomes from 50 (46.3%) individuals were successfully genotyped, including 25 of 36 (69.4%) from CHB and 25 of 72 (34.7%) from OBI. The low amplification rate likely reflects difficulties in amplifying longer fragments from individuals with low HBV levels such as occurs during OBI infections [64]. Twenty-seven samples were successfully genotyped from the whole 3.2 kb HBV genome (18 CHB and nine OBI) and 23 from the 3 kb fragment (seven CHB and 16 OBI). The circulating genotypes were 24 A1 (48%), 24 D3 (48%), and two E (4%), Supplementary Figure S1. For OBI participants, subgenotype A1 was found in 12 (48%) individuals, subgenotype D3 in 12 (48%), and genotype E in one (4%), with the same proportions found in CHB participants. OBI and CHB sequences clustered together (Figure 1 and Figure 2). There were three serotypes that were found in this study; *adw*2 was found in all genotype As; *ayw*2 was found in all genotype Ds, except one, which was *ay* (could not be serotyped further); and *ayw*4 was found in the genotype E isolate.

### 3.1. Results for Subgenotypes A1 and D3

Most sequences from this study grouped with the southern African A1, as shown in Figure 1. Only one sequence (MA94)—from an HBsAg positive participant—clustered with the Asian A1s. Mutations unique to MA94 were _preS2_A7T, _preS2_V17I, _preS2_T31I (PreS2), and _preS1_A90V. There was no separate clustering of HBV sequences from HBsAg-negative and HBsAg-positive participants. Most sequences from Botswana had the subgenotype A1 unique, as in the preS1 region (Q54, V74, A86 and V91) and in the preS2 region (L32) [70,71], except for three sequences with mutations at position 86 (two had _preS1_A86T and one was _preS1_A86V). When comparing the sequences in preS1 ORF (positions five, six, and 25) and preS2 (position 48), all Botswana sequences had 5S except MA101 and MA87, which were 5P. At position six, all were 6A except MA94, MA95, and MA85, which were 6S, as well as MA89, which was 6T. At position 25, however, all were 25F, except two, MA95, and MA85, which were 25L. In the PreS2 at position 48, all study sequences had 48R except MA95 and MA85, which had 48T. Moreover, in the PreS2 region at position 38, the majority of Botswana sequences had 38T except eight (MA94, MA101, MA89, MA87, MA98, MA95, MA86, and MA85), which had 38I. Additionally, all study sequences clustering with Zimbabwean HBV sequences had 38T. The Botswana genotype D3s clustered with South African D3s, Figure 2.

### 3.2. Nucleotide Divergence

Nucleotide pairwise distances for each ORF were calculated using Molecular Evolutionary Genetics Analysis (MEGA) version 7.0.26, Table 2 [72]. There was a statistically significant difference (*p* < 0.05) in the median nucleotide pairwise distances between CHB and OBI participants for most ORFs except in the preS2 (for both subgenotypes) and the X ORF (subgenotype D3), Table 2.

### 3.3. HBV Escape Mutations in the S ORF of OBI Versus CHB Sequences

Ten escape mutations associated with vaccine, diagnostic, or immunoglobulin therapy failure were identified in the S ORF. These include four mutations in OBI sequences (_S_K122R, _S_C124Y, _S_Q129H, and _S_K160N) and seven in CHB sequences (_S_P120S, _S_M103I, _S_Q129R, _S_G130N, _S_T140I, _S_G145R, and _S_K160N). One escape mutation—_S_K160N—occurred in both groups. The frequency of all mutations was one of 25 (4%) sequences, Table 3.

### 3.4. Occult-Unique Mutations

Occult-unique mutations were those mutations that only appeared in HBV sequences from OBI participants and not in CHB or reference sequences. For subgenotype A1, 12 HBV strains from OBI positive participants were compared with 119 CHB strains (12 CHB from this study + 107 GenBank CHB references). For subgenotype D3, 12 OBI strains were compared with 97 CHB strains (12 from this study + 85 from GenBank reference sequences). A total of 43 OBI unique mutations were identified in this study. Several of these mutations were reported previously. However, 26 (60.5%) were novel. Each mutation was found in a single strain, Table 4. The OBI unique mutations were found in HBV strains from 14 participants most of whom (12) harbored multiple mutations, Table 5.

### 3.5. Signature Amino Acid Analysis

The VESPA available at https://www.hiv.lanl.gov/content/sequence/VESPA/vespa.html was used to determine signature aa occurring in HBV sequences from OBI participants compared to CHB sequences. CHB sequences from the current study were used as background, and all analyses were performed by subgenotype. A total of 16 signature aa were detected, 12 in subgenotype A1 and four in subgenotype D3, Table 6. The PreS2 and C ORFs had no signature aa in either subgenotype.

### 3.6. Immune Selection Pressure

There were 26 negatively selected and no positively selected codons in the C ORF. Three of the negatively selected codons in the CHB group (four, 97, and 45) were in positions with OBI-unique mutations in the respective subgenotype. Two positions were under negative selection in both CHB and OBI participants (96, 122). None of the signature aa overlapped with codons under negative selection pressure. Five of the codons (four, seven, 68, 69, and 122) were within known HBV T cell epitopes: 1–20, 50–69, and 121–140. In the Pol ORF, there were 50 negatively selected codons and one positively selected codon. Only one OBI-associated mutation (rh81) overlapped with a codon under negative selection in the CHB participants, and two codons were negatively selected in both CHB and OBI participants. In the PreS1 and S region, two and one codons, respectively, were negatively selected in both CHB and OBI participants. In the Pre S1 region, seven of the codons were in HBV immune epitope positions: (28, 29) in the 21–30 T cell epitope (42, 44, 46) in the 29–48 T cell epitope and (85 and 90) in the 81–95 T cell epitope. In the X region, two codons were in epitopes positions (86 and 95): B cell epitope 85–110, whereas in the S region, one codon (148) was situated in a B cell epitope position: 122–148, Supplementary Table S1. There were significantly more negatively selected codons in the CHB than in the OBI (*p* = 0.031) sequences.

## 4. Discussion

This is the first study to report whole genome and nearly whole genome HBV sequences from Botswana and their molecular characterization. We also report the HBV genotypes in participants with OBI, and we identified mutations associated with OBI.

Subgenotypes A1, D3, and genotype E were identified in both OBI and CHB participants, which were also isolated from CHB participants in previous studies carried out in Botswana [4,80]. Most HBV sequences from Botswana clustered with other African sequences both at whole genome and at whole surface region level, as indicated in a study by Makondo et al. [81]. Similar to the aforementioned South African study, in which some isolates clustered with Asian sequences, one sequence also clustered with Asian sequences [81]. In further concordance with the South African study, sequences from HBsAg-positive and HBsAg-negative participants clustered together [81].

There are several mechanisms that have been implicated in OBI, including mutations in the HBV genome. However, there are few studies that have undertaken mutational analysis on nearly whole genomes of OBI strains owing to the difficulty in amplifying OBI samples with low viral loads [35,36,64,82,83,84]. Hence, most studies have focused on fragments of the HBV genome, especially the surface gene [41,42,64,85,86,87]. This is the first study to perform mutational analysis of nearly whole HBV genome sequences from Botswana.

The S ORF is evaluated most frequently for identification of OBI associated mutations. Some mutations in this ORF may lead to decreased detection by enzyme-linked immunosorbent assay (ELISA) kits, whereas some mutations demonstrated decreased secretion of HBsAg or increased retention of HBsAg [41,42,64,85,86,87]. All OBI associated mutations identified in the S ORF in this study (Table 4) have been reported previously [88,89,90,91,92]; however, the S region has been extensively studied [41,42,64,85,86,87]. The sL97P and sT114I mutations are located in an immunodominant region, the major hydrophilic region (position 99 to 169) [93]. Furthermore, the sN131K and sP217L mutations have been associated with diagnostic escape [89,94]. One of the mutations (sC124Y) has been reported and characterized in OBI sequences from blood donors in China and has been found to diminish both viral antigenicity and viral secretion [75]. There are multiple mutations that have been reported before in the same position and found to impact OBI phenotype in both a similar or different manner, e.g., (sC124R, sC124Y, and sC124A); sC124A was found to decrease extracellular HBsAg by decreasing viral secretion, whereas sC124R and sC124Y decreased antigenicity and viral secretion [75,76]. Cysteine forms disulfide bridges, which are critical in the formation of the HBsAg structure, hence, loss of cysteine might alter the structure and impact immunogenicity [95].

Conversely, for D3 isolates, there was only one OBI-associated mutation (sQ129H), which has been reported and functionally characterized before and found to decrease viral secretion [76]. Other studies have, however, reported OBI associated mutations, which have never been reported before, such as a study in South Africa [41]. However, there are differences between this study and the current study. In the South African study, most OBI mutations were from subgenotype A2, a subgenotype not present in the current study [41]. Another study in South Africa also identified three new OBI-associated mutations for genotype A participants (sY72H, sI82T, and sA128T), which were not observed in the current study [65].

In Botswana, there were no OBI-unique mutations in the PreS1 ORF for subgenotype A1 participants. The absence of OBI associated mutations has been reported before; for example, a study in Turkey found no OBI associated mutations in the S gene of female sex workers [96]. Some studies have reported deletions, which reduced HBsAg production in OBI participants, whereas other studies reported point mutations in this ORF [41,44,65,97]. The studies that reported point mutations concur with the present study, which reported one OBI-associated mutation in PreS1 for subgenotype D3. The _preS1_S78N mutation was novel. A different variant (_preS1_S78G) was observed at the same position in subgenotype A1 participants from South Africa [41].

There were two OBI-unique mutations in the PreS2 ORF of the subgenotype D3 sequences, as in other studies, which also found point mutations in this ORF [41,65,96,98]. A different variant (_preS2_F122L) was reported at the position of the two OBI associated mutations and has been linked with HCC [99,100]. The OBI-unique mutations in the current study are within the attachment region for human albumin (aa 17–28). Therefore, they may reduce HBV infectivity, since preS2 is responsible for mediating HBV attachment to hepatocytes [101]. Some studies documented deletions in the preS2 ORF, which may reduce HBsAg secretion [98,102]. The two mutations _preS2_F22P and _preS2_F22H were novel. The loss of phenylalanine, an aromatic amino acid, might impact protein structure, as aromatic aa play a role in protein structure stabilization [103].

The OBI associated mutations have also been described in the C ORF [22]. In the current study, there were 16 OBI-unique mutations in this ORF, and some of these mutations were located in functionally relevant regions. Three of these mutations (cD2A, cI3R, and cD4Y) spanned CD4^+^ T cell epitope (aa 1–20), and two other mutations (cE64K and cI59L) from this study were located within another CD4^+^ T cell epitope (aa 50–69) [104,105]. Furthermore, mutations cE117Stop and cR127H were also found in a CD4^+^ T cell epitope (aa 117–131) [104,105]. There is a mutation (cR127H), which was located in the CD4^+^ T cell epitope (aa 120–140) [104,105]. Some of the 16 OBI mutations in the core region have been reported before. For instance, cE64K has been reported in OBI-positive children born to HBsAg positive mothers in Iran [22], whereas cE46D was found in OBI-positive Chinese patients [35]. The aforementioned study [35] also reported different variants at the same residue as some of the OBI associated mutations (aa positions 26, 59, 74, 87, and 113) [35]. cV74N, cS87N, and cF97I have also been reported previously [106], with cF97I also detected in HBV from India [107]. Mutation cR127H has been shown to block capsid formation [108]. In the present study, there were 10 novel OBI-unique mutations in the core region: cS26P, cD32G, cP45S, cI59L, cD2A, cI13R, cD4Y, cW102V, cF103V, and cE117Stop. Position 102 plays a role in capsid assembly [109].

The X ORF is essential for transcription, hence, mutations in this ORF may affect viral replication [64]. In this study, there were seven OBI associated mutations identified in the X region, four of which have been previously reported. Mutation xS31A has been reported previously, and phosphorylation of the X protein occurs at serine 31 [110,111]. Mutations xQ87L, xS101P, and xL116V have also been reported in the literature [99,112]. In the present study, three novel OBI-unique mutations were detected in this ORF (xS11A, xV15I, and xP11S). Other studies in India reported OBI-associated mutations in the X ORF [113]. Some studies have reported deletions in the X region, which are linked to OBI. For instance, a Korean study documented an 8 bp deletion in OBI strains [36]. These deletions were shown to decrease HBsAg and HBV secretion and therefore may be responsible for the OBI phenotype [36]. In spite of this, in the present study, similar to other studies, there were no long stretches of deletions in the OBI strains [22].

Several studies have reported mutations in the Pol gene, which lead to drug resistance, and because of the overlapping nature, the mutations also simultaneously lead to changes in the S region, which may affect HBsAg production [114]. For example, studies in India and South Africa documented drug resistance mutations in OBI-positive participants [113,115]. In this study, rtT128I has also been reported similar to several studies including in chronic participants, and it has been linked to lamivudine drug resistance [116,117,118,119,120,121]. One of the mutations found in this study (spL140I) has been reported in HIV-infected participants in Germany but has not been characterized yet [89]. A study in China reported a 218 bp deletion in the Pol region of OBI positive adults, which might explain the low level of HBV viral load in the OBI participants [67]. In the current study, there were no deletions. On the other hand, the Chinese study found no OBI-associated mutations in the Pol region [44]. There were 10 novel OBI mutations in this region: tpN120Y, tpK155R, spW64R, spS91T, spP103S, spS133G, rtT225A, rtY257F, rtA329T, and rhI81M.

In most ORFs, more codons were under negative immune selection pressure in the CHB participants compared to the OBI participants. These results concur with a South African study, which found more negatively selected codons in the surface and Pol region in chronic participants compared to their OBI counterparts [41]. The reduced negative selection in the OBI participants might be due to the low HBV DNA, a characteristic of OBI [19,41]. Several of the negatively selected codons identified in this study are similar to those described in the South African study [41]. The overlap might be due to some similarities between the populations owing to the close proximity of the two countries. In contrast to the 16 negatively selected codons, which occurred in sites with OBI-unique mutations, in the current study only four were detected. These differences may be because the former study had more CHB participants (31 vs. 24) and also variations in HBV genotypes studied (A1 and A2 versus A1 and D3) [41,122]. Generally, there were also more negatively selected sites in genotype A compared to genotype D. Differences between genotypes even in mutations have been reported before [122]. Some of the codons under negative immune selection pressure were in known HBV epitopes [123,124,125,126,127]. Furthermore, nucleotide divergence was higher in the CHB group compared to the OBI group for most ORFs, Table 2, which concurs with other studies [113]. These differences in diversity may be due to immune pressure in the CHB group, as HBsAg triggers a greater immune response, thereby increasing the number of mutations arising, as the virus responds to evade the immune system. The low diversity seen in the OBI group may be due to the lack of HBsAg.

Several limitations of this study should be noted. The modest sample size may not reflect all aspects of HBV sequences circulating in Botswana or Southern Africa. Furthermore, the preC region was not analyzed, because it was not amplified in most participants. As most study participants were HIV-positive, findings from this study may not be generalizable to HBV mono-infected participants. Future HBV full-length studies on a larger sample size, including HBV mono-infected individuals, are warranted.

In conclusion, mutations in HBV from OBI participants in Botswana have been determined for the first time. Some of the OBI-unique mutations reported here have been functionally characterized before and found to impact HBsAg secretion/production and HBV virion secretion, and hence have an impact in the OBI phenotype. Other OBI mutations have been reported before, but their functional relevance is still unknown. In addition to these, there were also mutations that were found in this study that have not been reported previously. This study, therefore, has added to the body of knowledge of OBI-associated mutations by reporting novel OBI-unique mutations. Knowledge of OBI-unique mutations is important considering that with increased knowledge of OBI, diagnostic and preventative measures may be put in place in order to eliminate HBV per WHO goals.

## Figures and Tables

**Table 1:** Primers used for PCR and for sequencing.

Primer Name	Primer Sequence	Coordinates from *EcoR*I Site	Reference
P1	5′CCGGAAAGCTTGAGCTCTTCTTTTTCACCTCTGCCTAATCA 3′	1821–1841	[51]
P2	5′CCGGAAAGCTTGAGCTCTTCAAAAAGTTGCATGGTGCTGG 3′	1823–1806	[51]
**P3WRS**	**5′CTACTGTTCAAGCCTCCAAGC 3′**		[52]
**P4WRS**	**5′CGCAGACCAATTTATGCCTAC 3′**		[52]
**KU1**	**5′CATAAGAGGACTCTTGGACT 3′**	1653–1672	[54]
**KU2**	**5′AATGTCAACGACCGACCTTG 3′**	1679–1698	[54]
**MA3**	**5′GAAAGAAGTCAGAAGGCAAA 3′**	1973–1954	[53]
**HBV Z**	**5′AGCCCTCAGGCTCAGGGCATA 3′**	1179–1199	[55]
**HBV 3**	**5′CGTTGCCKDGCAACSGGGTAAAGG 3′**	2478–2455	[55]
**HBV M**	**5′GACACACTTTCCAATCAATNGG 3′**	2306–2287	[55]
**HBV P**	**5′TCATCCTCAGGCCATGCAGT 3′**	1292–1311	[55]
**HBV H**	**5′TATCAAGGAATTCTGCCCGTTTGTCCT 3′**	1767–1793	[55]
**HBV N**	**5′ACTGAGCCAGGAGAAACGGACTGAGGC 3′**	1991–1965	[55]
**Werle AS**	**5′CGTCAGCAAACACTTGGC 3′**	1175–1192	[56]
**CoreF**	**5′GTGTGGATTCGCACTCCT 3′**	2269–2287	[41]
**P6**	**5′GGCAGGTCCCCTAGAAGAAGAACT 3′**	2363–2386	[51]

The primers used for sequencing are those shown in bold.

**Table 2:** Comparison of the median nnucleotide pairwise distance (%) between chronic and occult infections by open reading frame and subgenotype.

ORF	CHB n = 12	OBI n = 12	*p*-Value	CHB n = 12	OBI n = 12	*p*-Value
	Subgenotype A1 (n = 24) Median (Q1, Q3)			Subgenotype D3 (n = 24) Median (Q1, Q3)		
**Pol**	2.8 (1.8, 3.4)	0.5 (0.0, 1.4)	<0.001	0.7 (0.4, 0.9)	0.4 (0.3, 0.5)	<0.001
**S**	1.2 (0.6, 2.1)	0.2 (0.1, 0.8)	<0.001	0.3 (0.1, 0.9)	0.1 (0.0, 0.3)	<0.001
**PreS1**	3.2 (2.0, 5.5)	0.9 (0.0, 2.7)	<0.001	1.1 (0.6, 1.1)	0.3 (0.0, 1.7)	0.001
**PreS2**	0.6 (0.0, 0.6)	0.0 (0.0, 1.2)	0.387	0.6 (0.0, 0.6)	0.0 (0.0, 1.2)	0.387
**X**	1.5 (1.1, 1.8)	11.8 (8.9, 23.3)	<0.001	11.5 (1.9, 13.4)	8.6 (2.0,11.5)	0.103
**Core**	4.0 (2.3, 5.8)	6.7 (2.8, 15.5)	<0.001	0.9 (0.5–2.7)	1.1 (0.7, 1.7)	<0.001

Abbreviations: CHB: Chronic hepatitis B; OBI: Occult hepatitis B infections; ORF: Open reading frame; Pol: Polymerase; S: Surface; PreS1: PreSurface 1; PreS2: PreSurface 2.

**Table 3:** HBV escape mutations in the S ORF of OBI versus CHB sequences.

Mutation	HBV Type	Reported Impact	References
_S_P120S	OBI	Vaccine, detection escape	[73]
_S_K122R	OBI	Decreased HBsAg expression, diagnostic escape	[42,74]
_S_C124Y	OBI	Immunoglobulin therapy escape, decreases antigenicity and viral secretion	[73,75]
_S_Q129H	OBI	Vaccine and immunoglobulin therapy escape, decreases viral secretion	[73,76]
_S_K160N	OBI/CHB	Decreases antigenicity and viral secretion	[77]
_S_M103I	CHB	Decreases antigenicity	[42]
_S_Q129R	CHB	Vaccine and detection escape and decrease viral secretion	[73,75]
_S_G130N	CHB	Diagnostic and vaccine escape	[78,79]
_S_T140I	CHB	Decreases viral secretion	[75]
_S_G145R	CHB	Vaccine, detection and immunoglobulin therapy escape, decreased antigenicity and viral secretion	[75]

Abbreviations: CHB: Chronic hepatitis B; OBI: Occult hepatitis B infections; S: Surface.

**Table 4:** OBI unique mutations in the different ORFs.

ORF/Region	Variant	Subgenotype
PreS1	_preS1_ **S78N**	D3
PreS2	_preS2_**F22P**, _preS2_**F22H**	D3
S	_s_L97P, _s_T114I, _s_C124Y, _s_N131K, _s_P217L	A1
S	_s_Q129H	D3
Pol	**_tp_N120Y**, **_tp_K155R**, **_sp_S91T**, **_sp_S133G**, _rt_L140I, **_rt_T225A**, **_rt_A329T**, **_rh_I81M**	A1
Pol	**_sp_W64R**, **_sp_P103S**, _rt_T128I, **_rt_Y257F**	D3
X	**_x_S11A**, **_x_V15I**	A1
X	**_x_P11S**, _x_S31A, _x_Q87L, _x_S101P, _x_L116V	D3
Core	**_c_S26P**, **_c_D32G**, **_c_P45S**, _c_E46D, **_c_I59L**, **_c_E117Stop**, _c_R127H	A1
Core	**_c_D2A**, **_c_I3R**, **_c_D4Y**, _c_E64K, _c_V74N, _c_S87N, _c_F97I, **_c_W102V**, **_c_F103V**	D3

Abbreviations: ORF: Open reading frame; S: surface; preS1: Pre-Surface 1; preS2: Pre-Surface 2; Pol: Polymerase; TP: Terminal protein; SP: spacer; RT: Reverse transcriptase; RH: RNase H; C: core. Novel OBI unique mutations are shown in bold.

**Table 5:** OBI unique mutations in different participants.

PID	Subgenotype	Variant
MA121	D3	_preS2_F22H
MA122	D3	_preS1_S78N, _preS_2F22P, _sp_W64R, _sp_P103S
MA123	D3	_c_D2A, _c_E64K
MA127	D3	_rt_T128I, _c_W102V, _c_F103V
MA128	D3	_x_P11S, _x_S31A, _x_S101P, _x_L116V, _c_V74N, _c_S87N, _c_F97I
MA125	D3	_c_I13R, _c_D4Y
MA107	A1	_s_N131K, _rt_L140I
MA98	A1	_s_L97P, _s_C124Y, _tp_N120Y, _tp_K155R, _x_V15I
MA103	A1	_s_P217L, _sp_S91T, _sp_S133G, _c_S26P, _c_D32G, _c_P45S, _c_E46D, _c_I59L
MA102	A1	_rt_T225A
MA99	A1	_c_E117Stop, _c_R127H
MA126	D3	_s_Q129H, _rt_Y257F, _x_Q87L
MA100	A1	_rh_I81M, _x_S11A
MA101	A1	_s_T114I, _rt_A329T

Abbreviations: S: surface; preS1: Pre-Surface 1; preS2: Pre-Surface 2; TP: Terminal protein; SP: spacer; RT: Reverse transcriptase; RH: RNase H; C: core.

**Table 6:** Signature amino acids found mostly in HBV sequences from OBI participants compared to sequences from CHB participants.

ORF	Sub Genotype	Signature Amino Acid	Number in OBI Participants *	Number in CHB Participants *
PreS1	D3	_preS1_P65L	11	5
PreS1	A1	_PreS1_P94T	9	6
Surface	A1	_SN131T_	7	4
Surface	A1	_S_A194V	10	5
Pol	D3	_rh_I54V	6	4
Pol	A1	_tp_H87Q	9	5
Pol	A1	_sp_T11A	9	5
Pol	A1	_sp_A68P	10	5
Pol	A1	_sp_S97H	9	5
Pol	A1	_rt_I103V	8	4
Pol	A1	_rt_Q139H	7	4
Pol	A1	_rt_S332N	10	5
Pol	A1	_rt_K333Q	10	5
Pol	A1	_rh_P2S	9	4
X	D3	_x_G27R	6	3
X	D3	_x_F30L	7	3

Abbreviations: ORF: Open reading frame; OBI: occult hepatitis B infections; CHB: chronic hepatitis B infections; Pol: Polymerase; tp: Terminal protein; sp: Spacer; rt: Reverse transcriptase; rh: RNase H; S: Surface * is n = 12.

## Supplementary Materials

The following are available online at http://www.mdpi.com/2073-4425/9/9/453/s1, Table S1: Immune selection pressure results determined using fixed effects likelihood in the DataMonkey Figure S1: A BEAST tree of full length HBV sequences from Botswana and reference sequences from GenBank.

## References

[1] WHO Hepatitis B. http://www.who.int/mediacentre/factsheets/fs204/en/.

[2] Stanaway J.D., Flaxman A.D., Naghavi M., Fitzmaurice C., Vos T., Abubakar I., Abu-Raddad L.J., Assadi R., Bhala N., Cowie B. (2016). The global burden of viral hepatitis from 1990 to 2013: Findings from the global burden of disease Study 2013. Lancet.

[3] WHO (2017). Global Hepatitis Report. https://www.afro.who.int/sites/default/files/2017-06/9789241565455-eng.pdf.

[4] Matthews P.C., Beloukas A., Malik A., Carlson J.M., Jooste P., Ogwu A., Shapiro R., Riddel L., Chen F., Luzzi G. (2015). Prevalence and characteristics of hepatitis B virus (HBV) coinfection among HIV-positive women in South Africa and Botswana. PLoS ONE.

[5] Patel P., Davis S., Tolle M., Mabikwa V., Anabwani G. (2011). Prevalence of hepatitis B and hepatitis C coinfections in an adult HIV centre population in Gaborone, Botswana. Am. J. Trop. Med. Hyg..

[6] Anderson M., Gaseitsiwe S., Moyo S., Thami K.P., Mohammed T., Setlhare D., Sebunya T.K., Powell E.A., Makhema J., Blackard J.T. (2016). Slow CD4+ T-Cell recovery in human immunodeficiency virus/hepatitis B virus-coinfected patients initiating truvada-based combination antiretroviral therapy in Botswana. Open Forum Infect. Dis..

[7] Wester C.W., Bussmann H., Moyo S., Avalos A., Gaolathe T., Ndwapi N., Essex M., MacGregor R.R., Marlink R. (2006). Serological evidence of HIV-associated infection among HIV-1-infected adults in Botswana. Clin. Infect. Dis..

[8] Seeger C., Mason W.S. (2000). Hepatitis B virus biology. Microbiol. Mol. Biol. Rev..

[9] Chen M., Sälberg M., Hughes J., Jones J., Guidotti L.G., Chisari F.V., Billaud J.N., Milich D.R. (2005). Immune tolerance split between hepatitis B virus precore and core proteins. J. Virol..

[10] Glebe D., Urban S., Knoop E.V., Cag N., Krass P., Grün S., Bulavaite A., Sasnauskas K., Gerlich W.H. (2005). Mapping of the hepatitis B virus attachment site by use of infection-inhibiting preS1 lipopeptides and tupaia hepatocytes. Gastroenterology.

[11] Pang R., Tse E., Poon R.T. (2006). Molecular pathways in hepatocellular carcinoma. Cancer Lett..

[12] Beck J., Nassal M. (2007). Hepatitis B virus replication. World J. Gastroenterol..

[13] Buti M., Rodriguez-Frias F., Jardi R., Esteban R. (2005). Hepatitis B virus genome variability and disease progression: The impact of pre-core mutants and HBV genotypes. J. Clin. Virol..

[14] Orito E., Mizokami M., Ina Y., Moriyama E.N., Kameshima N., Yamamoto M., Gojobori T. (1989). Host-independent evolution and a genetic classification of the hepadnavirus family based on nucleotide sequences. Proc. Natl. Acad. Sci. USA.

[15] Kramvis A., Kew M., François G. (2005). Hepatitis B virus genotypes. Vaccine.

[16] Norder H., Courouce A.M., Coursaget P., Echevarria J.M., Lee S.D., Mushahwar I.K., Robertson B.H., Locarnini S., Magnius L.O. (2004). Genetic diversity of hepatitis B virus strains derived worldwide: Genotypes, subgenotypes, and HBsAg subtypes. Intervirology.

[17] Tatematsu K., Tanaka Y., Kurbanov F., Sugauchi F., Mano S., Maeshiro T., Nakayoshi T., Miyakawa Y., Mizokami M. (2009). A genetic variant of hepatitis B virus divergent from known human and ape genotypes isolated from a Japanese patient and provisionally assigned to new genotype J. J. Virol..

[18] Kramvis A. (2014). Genotypes and genetic variability of hepatitis B virus. Intervirology.

[19] Raimondo G., Allain J.P., Brunetto M.R., Buendia M.A., Chen D.S., Colombo M., Craxì A., Donato F., Ferrari C., Gaeta G.B. (2008). Statements from the Taormina expert meeting on occult hepatitis B virus infection. J. Hepatol..

[20] Liu C.J., Lo S.C., Kao J.H., Tseng P.T., Lai M.Y., Ni Y.H., Yeh S.H., Chen P.J., Chen D.S. (2006). Transmission of occult hepatitis B virus by transfusion to adult and pediatric recipients in Taiwan. J. Hepatol..

[21] Hoofnagle J.H., Seeff L.B., Bales Z.B., Zimmerman H.J. (1978). Type B hepatitis after transfusion with blood containing antibody to hepatitis B core antigen. N. Engl. J. Med..

[22] Shahmoradi S., Yahyapour Y., Mahmoodi M., Alavian S.M., Fazeli Z., Jazayeri S.M. (2012). High prevalence of occult hepatitis B virus infection in children born to HBsAg-positive mothers despite prophylaxis with hepatitis B vaccination and HBIG. J. Hepatol..

[23] Hu L.P., Liu D.P., Chen Q.Y., Harrison T.J., He X., Wang X.Y., Li H., Tan C., Yang Q.L., Li K.W. (2015). Occult HBV Infection may be transmitted through close contact and manifest as an overt infection. PLoS ONE.

[24] Rai R.R., Mathur A., Udawat H.P., Nepalia S., NijHawan S., Mathud A. (2007). Prevalence of occult hepatitis B & C in HIV patients infected through sexual transmission. Trop. Gastroenterol..

[25] Singh S.P., Singh S.K., Misra B., Panigrahi M.K., Misra D. (2013). A prospective study of prevalence of occult HBV infection and assessment of risk factors for HBV transmission in persons with occult HBV infection. J. Clin. Exp. Hepatol..

[26] Blaich A., Manz M., Dumoulin A., Schüttler C.G., Hirsch H.H., Gerlich W.H., Frei R. (2012). Reactivation of hepatitis B virus with mutated hepatitis B surface antigen in a liver transplant recipient receiving a graft from an antibody to hepatitis B surface antigen- and antibody to hepatitis B core antigen-positive donor. Transfusion.

[27] Pollicino T., Squadrito G., Cerenzia G., Cacciola I., Raffa G., Craxi A., Farinati F., Missale G., Smedile A., Tiribelli C. (2004). Hepatitis B virus maintains its pro-oncogenic properties in the case of occult HBV infection. Gastroenterology.

[28] Raimondo G., Caccamo G., Filomia R., Pollicino T. (2013). Occult HBV infection. Semin. Immunopathol..

[29] Samal J., Kandpal M., Vivekanandan P. (2012). Molecular mechanisms underlying occult hepatitis B virus infection. Clin. Microbiol. Rev..

[30] Mak D., de Villiers C.B., Chasela C., Urban M.I., Kramvis A. (2018). Viral and non-viral risk factors associated with the development of hepatocellular carcinoma in black South Africans: 2000-2012. PLoS ONE.

[31] Blackberg J., Kidd-Ljunggren K. (2000). Occult hepatitis B virus after acute self-limited infection persisting for 30 years without sequence variation. J. Hepatol..

[32] Askari A., Hassanshahi G.H., Ghalebi S.R., Jafarzadeh A., Mohit M., Hajghani M., Arababadi M.K. (2014). Intensity of HLA-A2 Expression Significantly Decreased in Occult Hepatitis B Infection. Jundishapur J. Microbiol..

[33] Sagnelli E., Coppola N., Scolastico C., Filippini P., Santantonio T., Stroffolini T., Piccinino F. (2000). Virologic and clinical expressions of reciprocal inhibitory effect of hepatitis B, C, and delta viruses in patients with chronic hepatitis. Hepatology.

[34] Mphahlele M.J., Lukhwareni A., Burnett R.J., Moropeng L.M., Ngobeni J.M. (2006). High risk of occult hepatitis B virus infection in HIV-positive patients from South Africa. J. Clin. Virol..

[35] Huang F.Y., Wong D.K.H., Seto W.K., Zhang A.Y., Lee C.K., Lin C.K., Fung J., Lai C.L., Yuen M.F. (2014). Sequence variations of full-length hepatitis B virus genomes in Chinese patients with HBsAg-negative hepatitis B infection. PLoS ONE.

[36] Kim H., Gong J.R., Lee S.A., Kim B.J. (2015). Discovery of a novel mutation (X8Del) resulting in an 8-bp deletion in the hepatitis B virus X gene associated with occult infection in Korean vaccinated individuals. PLoS ONE.

[37] Zhang Z., Zhang L., Dai Y., Zhang Y., Li J., Li X. (2016). Occult hepatitis B virus infection: Influence of S protein variants. Virol. J..

[38] Kim H., Kim B.J. (2015). Association of preS/S mutations with occult hepatitis B virus (HBV) infection in South Korea: Transmission potential of distinct occult HBV variants. Int. J. Mol. Sci..

[39] Kim H., Lee S.A., Wom Y.S., Lee H.J., Kim B.J. (2015). Occult infection related hepatitis B surface antigen variants showing lowered secretion capacity. World J. Gastroenterol..

[40] Powell E.A., Boyce C.L., Gededzha M.P., Selabe S.G., Mphahlele M.J., Blackard J.T. (2016). Functional analysis of “a” determinant mutations associated with occult HBV in HIV-positive South Africans. J. Gen. Virol..

[41] Powell E.A., Gededzha M.P., Rentz M., Rakgole N.J., Selabe S.G., Seleise T.A., Mphahlele M.J., Blackard J.T. (2015). Mutations associated with occult hepatitis B in HIV-positive South Africans. J. Med. Virol..

[42] Martin C.M., Welge J.A., Rouster S.D., Shata M.T., Sherman K.E., Blackard J.T. (2012). Mutations associated with occult hepatitis B virus infection result in decreased surface antigen expression in vitro. J. Viral Hepat..

[43] Zoulim F., Locarnini S. (2009). Hepatitis B virus resistance to nucleos(t)ide analogues. Gastroenterology.

[44] Fang Y., Teng X., Xu W.Z., Li D., Zhao H.W., Fu L.J., Zhang F.M., Gu H.X. (2009). Molecular characterization and functional analysis of occult hepatitis B virus infection in Chinese patients infected with genotype C. J. Med. Virol..

[45] WHO Draft Global Health Sector Strategies. http://apps.who.int/gb/ebwha/pdf_files/WHA69/A69_32-en.pdf?ua=1.

[46] Yuan Q., Ou S.H., Chen C.R., Ge S.X., Pei B., Chen Q.R., Yan Q., Lin Y.C., Ni H.Y., Huang C.H. (2010). Molecular characteristics of occult hepatitis B virus from blood donors in Southeast China. J. Clin. Microbiol..

[47] Chaudhuri V., Tayal R., Nayak B., Acharya S.K., Panda S.K. (2004). Occult hepatitis B virus infection in chronic liver disease: Full-length genome and analysis of mutant surface promoter. Gastroenterology.

[48] Ryan K., Anderson M., Gyurova I., Ambroggio L., Moyo S., Sebunya T., Makhema J., Marlink R., Essex M., Musonda R. (2017). High rates of occult hepatitis B virus infection in HIV-positive individuals initiating antiretroviral therapy in Botswana. Open Forum Infect. Dis..

[49] Chaudhury S., Williams P.L., Mayondi G.K., Leidner J., Holding P., Tepper V., Nichols S., Magetse J., Sakoi M., Moabi K. (2017). Neurodevelopment of HIV-exposed and HIV-unexposed uninfected children at 24 months. Pediatrics.

[50] Mbangiwa T., Kasvosve I., Anderson M., Thami P.K., Choga W.T., Needleman A., Phinius B.B., Moyo S., Leteane M., Leidner J. (2018). Chronic and occult hepatitis B virus infection in pregnant women in Botswana. Genes.

[51] Gunther S., Li B.C., Miska S., Krüger D.H., Meisel H., Will H. (1995). A novel method for efficient amplification of whole hepatitis B virus genomes permits rapid functional analysis and reveals deletion mutants in immunosuppressed patients. J. Virol..

[52] Zahn A., Li C., Danso K., Candotti D., Owusu-Ofori S., Temple J., Allain J.P. (2008). Molecular characterization of occult hepatitis B virus in genotype E-infected subjects. J. Gen. Virol..

[53] Candotti D., Opare-Sem O., Rezvan H., Sarkodie F., Allain J.P. (2006). Molecular and serological characterization of hepatitis B virus in deferred Ghanaian blood donors with and without elevated alanine aminotransferase. J. Viral. Hepat..

[54] Kuwahara R., Kumashiro R., Murashima S., Ogata K., Tanaka K., Hisamochi A., Hino T., Ide T., Tanaka E., Koga Y. (2004). Genetic heterogeneity of the precore and the core promoter region of genotype C hepatitis B virus during lamivudine therapy. J. Med. Virol..

[55] Maponga T.G. (2012). An investigation of hepatitis B virus in antenatal women tested for human immunodeficiency virus, in the Western Cape Province of South Africa. Master’s Thesis.

[56] Werle B., Cinquin K., Marcellin P., Pol S., Maynard M., Trépo C., Zoulim F. (2004). Evolution of hepatitis B viral load and viral genome sequence during adefovir dipivoxil therapy. J. Viral. Hepat..

[57] Drummond A.J., Rambaut A. (2007). BEAST: Bayesian evolutionary analysis by sampling trees. BMC Evol. Biol..

[58] Lole K.S., Bollinger R.C., Paranjape R.S., Gadkari D., Kulkarni S.S., Novak N.G., Ingersoll R., Sheppard H.W., Ray S.C. (1999). Full-length human immunodeficiency virus type 1 genomes from subtype C-infected seroconverters in India, with evidence of intersubtype recombination. J. Virol..

[59] Poon A.F., Frost S.D., Pond S.L. (2009). Detecting signatures of selection from DNA sequences using Datamonkey. Methods Mol. Biol..

[60] Los Alamos National Laboratoy, VESPA. https://www.hiv.lanl.gov/content/sequence/VESPA/vespa.html.

[61] Bell T.G., Yousif M., Kramvis A. (2016). Bioinformatic curation and alignment of genotyped hepatitis B virus (HBV) sequence data from the GenBank public database. Springerplus.

[62] Bell T.G., Kramvis A. (2015). Bioinformatics tools for small genomes, such as hepatitis B virus. Viruses.

[63] Yousif M., Kramvis A. (2013). Genotype D of hepatitis B virus and its subgenotypes: An update. Hepatol. Res..

[64] Zhu H.L., Li X., Zhang Z.H. (2016). Genetic variation of occult hepatitis B virus infection. World J. Gastroenterol..

[65] Martin C.M., Welge J.A., Shire N.J., Rouster S.D., Shata M.T., Sherman K.E., Blackard J.T. (2010). Genomic variability associated with the presence of occult hepatitis B virus in HIV co-infected individuals. J. Viral Hepat..

[66] Panigrahi R., Biswas A., Datta S., Banerjee A., Chandra P.K., Mahapatra P.K., Patnaik B., Chakrabarti S., Chakravarty R. (2010). Anti-hepatitis B core antigen testing with detection and characterization of occult hepatitis B virus by an in-house nucleic acid testing among blood donors in Behrampur, Ganjam, Orissa in southeastern India: Implications for transfusion. Virol. J..

[67] Chen S.J., Zhao Y.X., Fang Y., Xu W.Z., Ma Y.X., Song Z.W., Teng X., Gu H.X. (2012). Viral deletions among healthy young Chinese adults with occult hepatitis B virus infection. Virus Res..

[68] Hass M., Hannoun C., Kalinina T., Sommer G., Manegold C., Günther S. (2005). Functional analysis of hepatitis B virus reactivating in hepatitis B surface antigen-negative individuals. Hepatology.

[69] Garcia-Montalvo B.M., Ventura-Zapata L.P. (2011). Molecular and serological characterization of occult hepatitis B infection in blood donors from Mexico. Ann. Hepatol..

[70] Kimbi G.C., Kramvis A., Kew M.C. (2004). Distinctive sequence characteristics of subgenotype A1 isolates of hepatitis B virus from South Africa. J. Gen. Virol..

[71] Bowyer S.M., van Staden L., Kew M.C., Sim J.G. (1997). A unique segment of the hepatitis B virus group A genotype identified in isolates from South Africa. J. Gen. Virol..

[72] Tamura K., Kumar S. (2002). Evolutionary distance estimation under heterogeneous substitution pattern among lineages. Mol. Biol. Evol..

[73] Echevarria J.M., Avellón A. (2006). Hepatitis B virus genetic diversity. J. Med. Virol..

[74] Yong-Lin Y., Qiang F., Ming-Shun Z., Jie C., Gui-Ming M., Zu-Hu H., Xu-Bing C. (2012). Hepatitis B surface antigen variants in voluntary blood donors in Nanjing, China. Virol. J..

[75] Huang C.H., Yuan Q., Chen P.J., Zhang Y.L., Chen C.R., Zheng Q.B., Yeh S.H., Yu H., Xue Y., Chen Y.X. (2012). Influence of mutations in hepatitis B virus surface protein on viral antigenicity and phenotype in occult HBV strains from blood donors. J. Hepatol..

[76] Kwei K., Tang X., Lok A.S., Sureau C., Tamako G., Li J., Wands J., Tong S. (2013). Impaired virion secretion by hepatitis B virus immune escape mutants and its rescue by wild-type envelope proteins or a second-site mutation. J. Virol..

[77] Wu C., Zhang X., Tian Y., Song J., Yang D., Roggendorf M., Lu M., Chen X. (2010). Biological significance of amino acid substitutions in hepatitis B surface antigen (HBsAg) for glycosylation, secretion, antigenicity and immunogenicity of HBsAg and hepatitis B virus replication. J. Gen. Virol..

[78] Ma Q., Wang Y. (2012). Comprehensive analysis of the prevalence of hepatitis B virus escape mutations in the major hydrophilic region of surface antigen. J. Med. Virol..

[79] Avellón A., Echevarria J.M. (2006). Frequency of hepatitis B virus ’a’ determinant variants in unselected Spanish chronic carriers. J. Med. Virol..

[80] Anderson M., Gaseitsiwe S., Moyo S., Wessels M.J.C., Mohammed T., Sebunya T.K., Powell E.A., Makhema J., Blackard J.T., Marlink R. (2015). Molecular characterisation of hepatitis B virus in HIV-1 subtype C infected patients in Botswana. BMC Infect. Dis..

[81] Makondo E., Bell T.G., Kramvis A. (2012). Genotyping and molecular characterization of hepatitis B virus from human immunodeficiency virus-infected individuals in southern Africa. PLoS ONE.

[82] Taffon S., Genovese D., Blasi M., Pierotti P., Degli Esposti A., Catone S., Chionne P., Pulimanti B., Candido A., Dettori S. (2014). HBV whole-genome mutation profile in HIV-1/HBV coinfected patients in a long-term follow-up study. Infection.

[83] Allain J.P., Belkhiri D., Vermeulen M., Crookes R., Cable R., Amiri A., Reddy R., Bird A., Candotti D. (2009). Characterization of occult hepatitis B virus strains in South African blood donors. Hepatology.

[84] Mondal R.K., Khatun M., Banerjee P., Ghosh A., Sarkar S., Santra A., Das K., Chowdhury A., Banerjee S., Datta S. (2017). Synergistic impact of mutations in Hepatitis B Virus genome contribute to its occult phenotype in chronic hepatitis C virus carriers. Sci. Rep..

[85] Qiu J., Quin B., Rayner S., Wu C.C., Pei R.J., Xu S., Wang Y., Chen X.W. (2011). Novel evidence suggests hepatitis B virus surface proteins participate in regulation of HBV genome replication. Virol. Sin..

[86] Wu C., Deng W., Deng L., Cao L., Qin B., Li S., Wang Y., Pei R., Yang D., Lu M. (2012). Amino acid substitutions at positions 122 and 145 of hepatitis B virus surface antigen (HBsAg) determine the antigenicity and immunogenicity of HBsAg and influence in vivo HBsAg clearance. J. Virol..

[87] Tian Y., Xu Y., Zhang Z., Meng Z., Qin L., Lu M., Yang D. (2007). The amino Acid residues at positions 120 to 123 are crucial for the antigenicity of hepatitis B surface antigen. J. Clin. Microbiol..

[88] Kim J.H., Jung Y.K., Joo M.K., Kim J.H., Yim H.J., Park J.J., Kim J.S., Bak Y.T., Yeon J.E., Byun K.S. (2010). Hepatitis B viral surface mutations in patients with adefovir resistant chronic hepatitis B with A181T/V polymerase mutations. J. Korean Med. Sci..

[89] Reuter S., Oette M., Wilhelm F.C., Beggel B., Kaiser R., Balduin M., Schweitzer F., Verheyen J., Adams O., Lengauer T. (2011). Prevalence and characteristics of hepatitis B and C virus infections in treatment-naive HIV-infected patients. Med. Microbiol. Immunol..

[90] Coppola N., Onorato L., Iodice V., Starace M., Minichini C., Farella N., Liorre G., Filippini P., Sagnelli E., de Stefano G. (2016). Occult HBV infection in HCC and cirrhotic tissue of HBsAg-negative patients: A virological and clinical study. Oncotarget.

[91] Jeantet D., Chemin I., Mandrand B., Zoulin F., Trepo C., Kay A. (2002). Characterization of two hepatitis B virus populations isolated from a hepatitis B surface antigen-negative patient. Hepatology.

[92] Amponsah-Dacosta E., Lebelo R.L., Rakgole J.N., Selabe S.G., Gededzha M.P., Mayaphi S.H., Powell E.A., Blackard J.T., Mphahlele M.J. (2015). Hepatitis B virus infection in post-vaccination South Africa: Occult HBV infection and circulating surface gene variants. J. Clin. Virol..

[93] Lazarevic I. (2014). Clinical implications of hepatitis B virus mutations: Recent advances. World J. Gastroenterol..

[94] Gerlich W.H., Glebe D., Schüttler C.G. (2007). Deficiencies in the standardization and sensitivity of diagnostic tests for hepatitis B virus. J. Viral. Hepat..

[95] Ireland J.H., O’Donnell B., Basuni A.A., Kean J.D., Wallace L.A., Lau G.K., Carman W.F. (2000). Reactivity of 13 in vitro expressed hepatitis B surface antigen variants in 7 commercial diagnostic assays. Hepatology.

[96] Pinarbasi B., Onel D., Cosan F., Akyuz F., Dirlik N., Cakaloglu Y., Badur S., Besisik F., Demir K., Okten A. (2009). Prevalence and virological features of occult hepatitis B virus infection in female sex workers who work uncontrolled in Turkey. Liver Int..

[97] Sengupta S., Rehman S., Durgapal H., Acharya S.K., Panda S.K. (2007). Role of surface promoter mutations in hepatitis B surface antigen production and secretion in occult hepatitis B virus infection. J. Med. Virol..

[98] Kim H., Lee S.A., Kim D.W., Lee S.H., Kim B.J. (2013). Naturally occurring mutations in large surface genes related to occult infection of hepatitis B virus genotype C. PLoS ONE.

[99] Pollicino T., Raffa G., Costantino L., Lisa A., Campello C., Squadrito G., Levrero M., Raimondo G. (2007). Molecular and functional analysis of occult hepatitis B virus isolates from patients with hepatocellular carcinoma. Hepatology.

[100] Gopalakrishnan D., Keyter M., Shenoy K.T., Leena K.B., Thayumanavan T., Thomas V., Vinayakumar K.R., Panackel C., Korah A.T., Nair R. (2013). Hepatitis B virus subgenotype A1 predominates in liver disease patients from Kerala, India. World J. Gastroenterol..

[101] Hao Z., Zheng L., Kluwe L., Huang W. (2012). Ferritin light chain and squamous cell carcinoma antigen 1 are coreceptors for cellular attachment and entry of hepatitis B virus. Int. J. Nanomed..

[102] Pollicino T., Cacciola I., Saffoti F., Raimondo G. (2014). Hepatitis B virus PreS/S gene variants: Pathobiology and clinical implications. J. Hepatol..

[103] Burley S.K., Petsko G.A. (1985). Aromatic-aromatic interaction: A mechanism of protein structure stabilization. Science.

[104] Datta S., Ghosh A., Dasgupta D., Gosh A., Roychoudhury S., Roy G., Das S., Gupta S., Basu K., Basu A. (2014). Novel point and combo-mutations in the genome of hepatitis B virus-genotype D: Characterization and impact on liver disease progression to hepatocellular carcinoma. PLoS ONE.

[105] Zhand S., Tabarraei A., Nazari A., Moradi A. (2017). Cytotoxic T lymphocytes and CD4 epitope mutations in the pre-core/core region of hepatitis B virus in chronic hepatitis B carriers in Northeast Iran. Indian J. Gastroenterol..

[106] Berke J.M., Tan Y., Verbinnen T., Dehertogh P., Vergauwen K., Vos A., Lenz O., Pauwels F. (2017). Antiviral profiling of the capsid assembly modulator BAY41-4109 on full-length HBV genotype A-H clinical isolates and core site-directed mutants in vitro. Antivir. Res..

[107] Banerjee A., Banarjee S., Chowdhury A., Santra A., Chowdhury S., Roychowdhury S., Panda C.K., Bhattacharya S.K., Chakravarty R. (2005). Nucleic acid sequence analysis of basal core promoter/precore/core region of hepatitis B virus isolated from chronic carriers of the virus from Kolkata, eastern India: Low frequency of mutation in the precore region. Intervirology.

[108] Ponsel D., Bruss V. (2003). Mapping of amino acid side chains on the surface of hepatitis B virus capsids required for envelopment and virion formation. J. Virol..

[109] Wu S., Zhao Q., Zhang P., Kulp J., Hu L., Hwang N., Zhang J., Block T.M., Xu X., Du Y. (2017). Discovery and Mechanistic Study of Benzamide Derivatives That Modulate Hepatitis B Virus Capsid Assembly. J. Virol..

[110] Utama A., Purwantomo S., Siburian M.D., Dhenni R., Gani R.A., Hasan I., Sanityoso A., Miskad U.A., Akil F., Yusuf I. (2009). Hepatitis B virus subgenotypes and basal core promoter mutations in Indonesia. World J. Gastroenterol..

[111] Khattar E., Mukherji A., Kumar V. (2012). Akt augments the oncogenic potential of the HBx protein of hepatitis B virus by phosphorylation. FEBS J..

[112] Kim H., Jee Y.M., Song B.C., Hyun J.W., Mun H.S., Kim H.J., Oh E.J., Yoon J.H., Kim Y.J., Lee H.S. (2007). Analysis of hepatitis B virus quasispecies distribution in a Korean chronic patient based on the full genome sequences. J. Med. Virol..

[113] Saha D., Pal A., Sarkar N., Das D., Blackard J.T., Guha S.K., Saha B., Chakravarty R. (2017). Occult hepatitis B virus infection in HIV positive patients at a tertiary healthcare unit in eastern India. PLoS ONE.

[114] Sheldon J., Soriano V. (2008). Hepatitis B virus escape mutants induced by antiviral therapy. J. Antimicrob. Chemother..

[115] Selabe S.G., Lukhwareni A., Song E., Leeuw Y.G., Burnett R.J., Mphahlele M.J. (2007). Mutations associated with lamivudine-resistance in therapy-naive hepatitis B virus (HBV) infected patients with and without HIV co-infection: Implications for antiretroviral therapy in HBV and HIV co-infected South African patients. J. Med. Virol..

[116] Mahabadi M., Norouzi M., Alavian S.M., Samimirad K., Azad T.M., Saberfar E., Mahmoodi M., Ramezani F., Karimzadeh H., Malekzadeh R. (2013). Drug-related mutational patterns in hepatitis B virus (HBV) reverse transcriptase proteins from Iranian treatment-naive chronic HBV patients. Hepat. Mon..

[117] Vincenti D., Solmone M., Garbuglia A.R., Iacomi F., Capobianchi M.R. (2009). A sensitive direct sequencing assay based on nested PCR for the detection of HBV polymerase and surface glycoprotein mutations. J. Virol. Methods..

[118] Panigrahi R., Biswas A., De B.K., Chakrabarti S., Chakravarty R. (2013). Characterization of antiviral resistance mutations among the Eastern Indian Hepatitis B virus infected population. Virol. J..

[119] Santantonio T., Fasano M., Durantel S., Barraud L., Heichen M., Guastadisegni A., Pastore G., Zoulim F. (2009). Adefovir dipivoxil resistance patterns in patients with lamivudine-resistant chronic hepatitis B. Antivir. Ther..

[120] Ciftci S., Keskin F., Cakiris A., Akyuz F., Pinarbasi B., Abaci N., Dincer E., Badur S., Kaymakogly S., Ustek D. (2014). Analysis of potential antiviral resistance mutation profiles within the HBV reverse transcriptase in untreated chronic hepatitis B patients using an ultra-deep pyrosequencing method. Diagn. Microbiol. Infect. Dis..

[121] Villet S., Pichoud C., Villeneuve J.P., Trépo C., Zoulim F. (2006). Selection of a multiple drug-resistant hepatitis B virus strain in a liver-transplanted patient. Gastroenterology.

[122] Sunbul M. (2014). Hepatitis B virus genotypes: Global distribution and clinical importance. World J. Gastroenterol..

[123] Stemler M., Weimer T., Tu Z.X., Wan D.F., Levrero M., Jung C., Pape G.R., Will H. (1990). Mapping of B-cell epitopes of the human hepatitis B virus X protein. J. Virol..

[124] Jung M.C., Diepolder H.M., Pape G.R. (1994). T cell recognition of hepatitis B and C viral antigens. Eur. J. Clin. Investig..

[125] Mizukoshi E., Sidney J., Livingston B., Ghany M., Hoofnagle J.H., Sette A., Rehermann B. (2004). Cellular immune responses to the hepatitis B virus polymerase. J. Immunol..

[126] Zu Putlitz J., Landford R.E., Carlson R.I., Notvall L., de la Monte S.M., Wands J.R. (1999). Properties of monoclonal antibodies directed against hepatitis B virus polymerase protein. J. Virol..

[127] Lin Y.M., Jow G.M., Mu S.C., Chen B.F. (2013). Naturally occurring hepatitis B virus B-cell and T-cell epitope mutants in hepatitis B vaccinated children. Sci. World J..

