# Supplementary material for: Molecular Characterization of Near Full-Length Genomes of Hepatitis B Virus Isolated from Predominantly HIV Infected Individuals in Botswana
Source: Genes (Basel). 2018 Sep 7;9(9):453. doi: 10.3390/genes9090453 (PMC6162474; doi:10.3390/genes9090453)
Supplement: Supplementary file 1 [file genes-09-00453-s001.pdf]

Table S1. Immune selection pressure results determined using fixed effects likelihood in the DataMonkey.

ORF	Sub-genotype	Type of HBV	Positively selected codon position	Negatively selected codon positions					
Core	D	OBI	None	29	30	43	48	68	90
				92	96	107	145		
Core	D	CHB	None	**4**	**97**	117			
Core	A	OBI	None	48	96#	122 #			
Core	A	CHB	None	7	34	43	**45**	69	96#
				99	114	122 #	145		
Pol	D	OBI	None	rt241	rt318	rh37	rh49	rh65	rh69
Pol	D	CHB	None	tp47	rt32	rt142	rh30	rh66	rh100
Pol	A	OBI	None	tp106	tp132	sp95	rt241#	rt281	rh20#
Pol	A	CHB	rt271	tp15	tp48	tp64	tp66	tp69	tp80
				tp95	tp109	tp132	tp144	tp157	tp171
				rt241#	rt249	rt251	rt259	rt279	rt282
				rt286	rt307	rt317	rt320	rt334	rh19
				rh20#	rh26	rh43	rh56	rh58	rh81
				rh92	rh101				
X	D	OBI	none	86					
X	D	CHB	none	80					
X	A	OBI	none	95					

S	A	OBI	none	101 #					
S	A	CHB	none	101 #	148				
PreS 1	D	OBI	none	70					
PreS 1	D	CHB	none	28					
PreS 1	A	OBI	none	7#	20	42#	85		
PreS 1	A	CHB	90	7#	29	42#	44	46	56
PreS 2	A	OBI	none	10					

Immune selection pressure was determined in codons using fixed effects likelihood in the DataMonkey. ORF: open reading frame; HBV: hepatitis B virus; OBI: occult hepatitis B infections; CHB: chronic hepatitis B infections; Pol: Polymerase; S: Surface; PreS1: pre-surface1; PreS2: pre-surface2. The symbol # depicts codons which are under negative selection pressure in both OBI and CHB participants. Codons corresponding to OBI associated mutations positions are shown in bold.

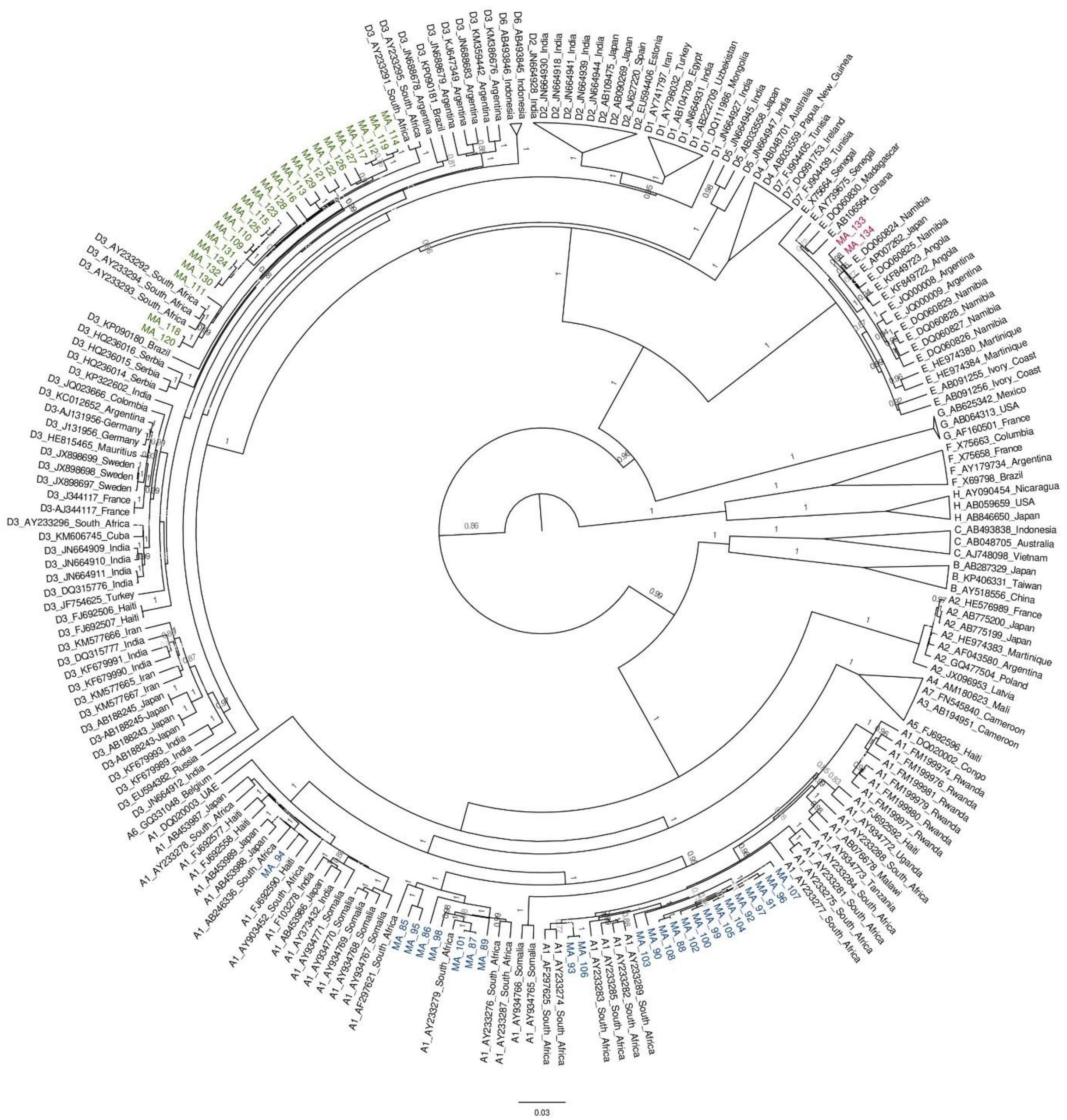

Figure S1. A phylogenetic tree of the nearly whole genome hepatitis B virus (HBV) sequences generated by Bayesian Evolutionary Analysis by Sampling Trees (BEAST). Subgenotype A1 sequences from Botswana are highlighted in blue, subgenotype D3 sequences in green, and genotype E sequences in red. Black sequences represent GenBank references named by their subgenotype, accession number, and country of origin.
